# Typical performance of elderlypatients with Alzheimer disease on the Wisconsin Card Sorting Test (WCST)

**DOI:** 10.1590/s1980-57642008dn10200011

**Published:** 2007

**Authors:** José Humberto Silva-Filho, Sonia Regina Pasian, Francisco de Assis Carvalho do Vale

**Affiliations:** 1 Psychologist, Doctor in Psychology, Teacher at the Department of Psychology at the Federal University of Amazonas (CAPES).; 2 Psychologist, Doctor in Psychology, Teacher at the Department of Psychology and Education at the Faculty of Philosophy, Sciences and Letters of Ribeirão Preto, University of São Paulo.; 3 Neurologist, HCFMRP Behavioral Neurology Group Coordinator, Teacher on the Post-Graduation Program in Neurology/Neurosciences – FMRP-USP.

**Keywords:** Alzheimer's disease, neuropsychological assessment, Wisconsin Card Sorting Test, executive functions, memory disorder, elderly people

## Abstract

The Wisconsin Card Sorting Test (WCST) is a neuropsychological assessment tool designed to assess executive functions, frequently used in cases of cognitive disorders. However, Brazilian neuroscientific settings lack standardization studies of psychological assessment instruments, especially in the neuropsychological area. Thus, the assessment of clinical groups including dementias and particularly Alzheimer's disease (AD) patients, may be compromised by the lack of analytical references.

**Objective:**

To characterize the performance of elderly patients with Alzheimer's disease on the WCST, aiming at establishing preliminary evaluative norms.

**Method:**

Thirty-six elderly patients (mean age of 75.8 years) with mild AD from a teaching hospital were assessed using the printed form of the WCST.

**Results:**

The elderly patients with AD had impaired performance on the various WCST technical indicators, highlighting cognitive deficit with traces of stereotyped behavior and failures in working memory, conceptualization and learning. The results allowed preliminary norms to be defined for elderly AD patients on the various WCST indicators, grading their performance in eight diagnostic areas and yielding the identification of different levels of impairment of executive functions in these elderly patients.

**Conclusions:**

The results demonstrated specific aspects of performance on the WCST by elderly people with AD, highlighting the effect of the disease on cognitive performance and executive functioning. Those normative references, although preliminary make a significant contribution to the neuropsychological assessment of AD patients in the Brazilian context, within the informative scope of the WCST.

## Abstract (pt)

O Teste Wisconsin de Classificação de Cartas (Wisconsin Card Sorting Test - WCST) é um instrumento de avaliação neuropsicológica voltado para o exame das funções executivas, freqüentemente adotado em casos de transtornos cognitivos. No entanto, a realidade brasileira carece de estudos de padronização de instrumentos de avaliação psicológica, em especial na área neuropsicológica. Desta forma, a avaliação de grupos clínicos pode ficar comprometida por ausência de referenciais analíticos, atingindo avaliação das demências e, em particular, a Doença de Alzheimer (DA).

**Objetivo:**

Caracterizar o desempenho típico de idosos com DA no WCST, visando a elaboração de normas avaliativas preliminares.

**Método:**

Foram avaliados, por meio da versão impressa do WCST, 36 idosos em fase inicial de DA, voluntários, de ambos os sexos, com idade média de 75,8 anos, pacientes ambulatoriais em hospital-escola de Ribeirão Preto (SP).

**Resultados:**

Os idosos com DA alcançaram reduzido desempenho nos diferentes indicadores técnicos do WCST, apontando prejuízo cognitivo, com sinais de comportamento estereotipado e falhas na memória operativa, na formação de conceitos e na aprendizagem. Os resultados permitiram elaboração de normas preliminares para estes idosos com DA nos diversos indicadores do WCST, classificando seu desempenho em oito faixas diagnósticas, permitindo identificação de níveis de comprometimento das funções executivas em idosos.

**Conclusões:**

As atuais evidências empíricas apontaram especificidades de produção no WCST em idosos, apontando influência de DA no desempenho cognitivo e no funcionamento executivo. Estes referenciais normativos, mesmo que preliminares, trazem importante contribuição à avaliação neuropsicológica de DA no contexto brasileiro, dentro das possibilidades informativas do WCST.

The rise of populational longevity seen in recent decades is associated to a higher incidence of dementia-related-syndromes and disorders worldwide, revealing a concerning situation which public health policies must address.^1^ Many studies in a range of areas have focused on identifying predisposing and risk factors for aging-related diseases, as well as complications associated to the development process in this age bracket. The area remains largely unknown, calling for further investigation.^2^

Demential syndromes are clinical pictures which, in general, are directly associated to aging. The more age increases, the greater the risk for such syndromes. Their prevalence ranges from 0.7% in the 60 to 64-year-old bracket, and doubles in geometrical progression every five years thereafter, reaching 38.6% in the 90-95 years old age range.^3^

According to the DSM-IV^4^, demential syndromes are characterized by mnemonic function impairments, for both learning new things and recalling previously learned content. In order to correctly diagnose dementia, signs of memory impairment are investigated, such as aphasia, apraxia or agnosia, and chiefly, executive functioning disturbances. Indicative signs of dementia are characterized by cognitive deterioration, behavioral changes and significant social and occupational impairment in the individual’s functioning.^5^

Alzheimer Disease (AD) is the most frequent dementia, with an average prevalence of 55%.^6,7^ This is a neurological disease consisting of progressive neural loss and the presence of senile plaques and neurofibrillar bundles in the brain.^8^ As the diagnosis of AD is possible only by observing these histopathological alterations through biopsies or necropsies, such diagnoses are eminently clinical and based on the exclusion of other possible causes for the demential picture.^9^ In general, the first indicators of AD are cognitive complaints, particularly those related to memory. Moreover, precociously, executive function impairments are also worth of note.^10-12^

According to Papazian, Alfonso and Luzondo,^13^ executive functions are mental processes which intentionally solve internal problems (representations of creative activities, conflicts related to social interaction, communication and motivation), and so as external ones, resulting from the interaction between the individual and his or her surroundings. The aim of executive functions is to efficiently and adequately solve these reality-related challenges, either in individual or social aspects. Although very complex, the concept of executive functions is often used to represent a plurality of individual resources set in the adaptative process, resulting in higher or lower functionality.

The cognitive impairments associated to the executive functions in AD and other dementia include disorders in initiative-taking ability, motivation, aim formulation, behavior planning and self-control, all associated to pre-frontal cortex damage.^14^ Individuals show gradual and severe losses in their general adaptative functionality and abstraction ability, albeit potentially retaining good resources in terms of adaptation. The complexity of these psychic functions demands broad and in-depth investigation, stressing the importance of adequate technical care in achieving correct diagnosis and therapeutic planning of cases.

Among the resources available to Psychology professionals, the *Wisconsin Card Sorting Test (WCST)*^15-19^ has been a widely referenced instrument in international literature for the assessment of executive functions, showing consistent illustrative studies of its construct and discriminative validity, attaining significant repercussions in the scientific milieu. One study evidencing the WCST’s positive technical aspects was the meta-analysis of Demakis^20^, who examined 24 articles on this instrument, published from 1963 to 2001, involving a total of 1349 individuals. In this study, the author compared WCST results of 644 brain-damaged patients with 705 non-frontal-damaged individuals. The results showed WCST’s sensitivity for discriminating individuals with executive malfunctions associated to frontal lobe damage, strengthening the informative possibilities of this psychological assessment instrument.

In regard to the WCST use in the clinical context of dementia, Vost^21^ identified clear evidences of executive function impairments in patients with AD, compared with healthy individuals, using the WCST. Glozman^22^ also identified that scores for some WCST indicators are sufficiently sensitive to discriminate elderly persons with memory impairments related to aging from elderly persons with previously diagnosed AD. However, scores of the WCST for “Perseverative Responses” in Glozman’s study were similarly poor in both groups. Such results suggest a global intellectual decay in elderly persons, which can be associated to the aging process itself, resulting form short-term memory failures and constructional skills mediating the executive functions. Difficulties in memory, according to Glozman, could already indicate a cognitive disorder, preceding AD’s development. Thus, this asymptomatic stage of the disease, characterized by low cognitive impairment, can lead to development of AD, depending on the combination of several internal and external factors that could be at play, unbeknown to doctors or family members^9^. Therefore, swift identification of suggestive signs of dementia would be imperative in order to offer better therapeutic interventions for the patient, as well as provide them with increased benefits and better quality of life.^23^ The WCST’s informative capabilities lie within this wide spectrum, given its international acknowledgement as a valid technique for this kind of psychological investigation.

The WCST was developed in 1948 by Grant and Berg,^15,16^ at the University of Wisconsin, causing an important repercussion in the analysis of abstract behaviors associated to the executive functions. Over the 50 years since, a profusion of different versions of this instrument has emerged. In 1981, Robert K. Heaton published the first handbook of the test entitled *Psychological Assessment Resources*.^17^ In 1993, Heaton, Chelune, Talley, Kay and Curtiss published a revised and broader second edition of this handbook, in which an adequate standardization of the WCST was presented.^18^ This methodological review was able to merge 53 different identified technical models for the instrument, considering its structure, management and results interpretation.^18^ This version of the WCST’s handbook has been considered the most advanced and reliable so far, being the most cited in international literature on the area since its publication.^24^ This handbook offers an operationally precise description of the WCST’s application method, results codification and interpretation. In Brazil, this version of the instrument was published in 2005, by Casa do Psicólogo Press, along with studies of the Brazilian adaptation, standardization and normalization by Jurema Alcides Cunha’s team.^19^ However, the Brazilian norms published to date cover 6 to 18-year-olds only, representing just the beginning of these studies in Brazil. New studies based on the WCST are currently being conducted for other age^25^ and clinical groups, such as bipolar mood disorder or obsessive-compulsive disorder patients, alcohol and drug abusers, homicide persons and patients with temporal lobe epilepsy. Considering the elderly, publication of a Brazilian handbook exclusively for old-aged clinical groups (Major Depression disorder, Parkinson Disease and Alzheimer Disease) in the near future.^26^ Such ongoing studies, including the present work, adopt the technical standardization of the WCST’s Brazilian edition by Heaton et al,^19^ adapted for the sociocultural reality of Brazil.

The WCST consists of a psychological test that presents a problem (classifying cards based on standard stimuli) with many possible solutions. It requires adaptation and updating of mental strategies based on the assessor’s feedback during the task. The test is composed of stimuli-cards and response-cards. Four of them are shown to the subject as standard stimuli and two 64-card packs are used as responses. The cards have to be classified according to standard stimuli, one at a time. The subjects receives feedback from the assessor (“right” or “wrong” classification) after each response-card is used. Classification criteria are not provided in the instructions, for the test aims to assess the subject’s skill in mentally formulating a hypothesis (concept) in order to solve the problem. Throughout the test, card classification criteria vary among considering “color (C)”, “form (F)” or “number (N)”, without warning the subject of these changes. This is done in order to verify, besides concept formation, abilities such as keeping focused on the task (working memory), inhibiting perseverative behaviors, behavior self-monitoring, planning skill, cognitive flexibility and task learning. The test concludes when the subject is able to correctly classify response-cards into six categories (color, form, number, color, form, number) in any number of trials or when all 128 cards have been used.^19^

There is no single global score as an assessment standard for the WCST. The results are presented in 16 assessment indicators, 13 of them with raw scores converted into normalized grades, namely: “Total Number of Errors”, “Percent Errors”, “Perseverative Responses”, “Percent Perseverative Responses”, “Perseverative Errors”, “Percent Perseverative Errors”, “Nonperseverative Errors”, “Percent Nonperseverative Errors”, “Percent Conceptual Level Responses”, “Number of Categories Completed”, “Trials to Complete First Category”, “Failure to Maintain Set”, “Learning to Learn”.

According to Heaton et al.,^18,19^ the diversity of assessment indicators in the WCST is one of its advantages compared to other instruments assessing executive functions. Besides offering a global evaluation of these functions, the WCST allows specific difficulties to be checked. Such dysfunctional areas could also be separately assessed through other specific WCST indicators. In this approach, both planning skill and cognitive flexibility are assessed through total success in the test. “Failure to Maintain Set” evaluates working memory, while behavior self-monitoring is examined through “total number correct”, “conceptual level responses” and “total number of errors”. Concept formation can be drawn from “trials to complete first category” and “number of categories completed” indicators. Inhibition of perseverative responses is assessed using all four indicators related to “perseverative responses”. Lastly, task learning is shown by the “learning to learn” indicator.^25^

According to WCST’s standardization,^18,19^ the interpretation of the results is based on percentile grades, T- and Standard scores, which are obtained from the raw grades in the test, in each assessment indicator, compared to normative groups. These positions are categorized into eight diagnostic ranges (Table 1), thus offering good informative and interpretative capabilities in the test.

**Table 1:** Diagnostic ranges and standardized scores of position measures in the WCST^18,19^.

Position measures				
Diagnostic range	Diagnostic categories	Percentile	T-score	Standard score
1	Above-average range	≥68	≥55	≥107
2	Average range	29-67	45-54	92-106
3	Below-average range	15-28	40-44	85-91
4	Mildly impaired range	6-14	35-39	77-84
5	Mildly-to-moderately impaired range	3-5	30-34	70-76
6	Moderately impaired range	2	25-29	62-69
7	Moderately-to-severely impaired range	1	20-24	55-61
8	Severely impaired range	<1	≥19	≥54

Given the WCST’s international importance in the neuropsychological assessment field, particularly for executive function investigation, the importance of further, in-depth studies into its suitability for individuals in the Brazilian sociocultural context is clear. Thus, the present work aimed to evaluate the WCST performance patterns in a sample of elderly persons in initial stages of AD, treated in an outpatient unit of a teaching-hospital in Ribeirão Preto (SP). Thus, the intention of this work was to develop preliminary normative references for the WCST in order to assess this elderly clinical group in the Brazilian context.

## Methods

Data was collected during psychological assessment of elderly patients attending the Behavioral Neurology Outpatient Unit (BNOU) at the Medical School of Ribeirão Preto’s Clinicas Hospital (Hospital das Clínicas da Faculdade de Medicina de Ribeirão Preto, HCFMRP), University of São Paulo (Universidade de São Paulo, USP). The institution Ethics Committee approved the research project (Process 15.407/2005). The BNOU is a tertiary outpatient unit that receives patients from other public health units referred by neurologists or psychiatrists, where patients attended display neurological and behavioral symptoms or complaints.^27^ More than half of all 1459 patients attended to date showed demential pictures. Differential diagnoses are made by the medical staff through a revised Routine Procedures Handbook protocol, developed in the BNOU. This protocol recommends, as part of the diagnosis process, a detailed anamnesis, general physical and neurological exam, besides annual neuroimage exams for monitoring. Also, the protocol orients medical procedures in cognitive-behavioral exams, used to investigate cognitive function disorders including memory, praxias, gnosias, calculation, language, abstraction and thought, as well as functional activity loss.^28,29^ For AD, worldwide-accepted criteria^30^ are used for diagnosis. Dementia progress is monitored using tests such as the Mini Mental State Examination (MMSE)^31^ and the Clinical Dementia Rating (CDR), associated to eminently descriptive, ecologically-oriented clinical judgment, which takes not only cognitive testing objective scores into consideration, but also the individual’s overall functionality in his or her daily life.^33^

Thirty-six BNOU patients participated in the present study, governed by the following inclusion conditions:

a) fulfilling of diagnosis requirements for AD;
b) presenting the initial stage of the disease, that is, having a CDR score from 0.5 to 1.0;
c) free to take part in the research (with family or guardian consent), signing a Term of Free and Informed consent;
d) not presenting a history of drug use/abuse;
e) not having sensorial or motor impairment preventing psychological assessment;
f) aged 60 years or older.

A total of 36 initial-AD-stage patients met these criteria for inclusion, with ages ranging from 62 to 86 years’ old (M=75.8 and SD=6.8). Most of the participants were female (63.9%) and educational level varied from 1 to 12 years of schooling (M=4.9 and SD=3.4). MEEM grades varied from 7-30 (M=18.31 and SD=4.60) suggesting dementia, although false negative results were also present. Considering the CDR, 19.4% of the participants had a score 0.5, and the remainder (80.6%) had a score of 1.0.

Data were collected from March to July, 2006, drawn from clinical records. Subsequently, subjects were submitted to the WCST (printed, full version) in a single session of approximately 1 hour, according to Brazilian adaptation and standardization.^19^ Results for the WCST’s technical indicators were calculated using specific software,^34^ in order to control for measurement accuracy.

The construction of normative standards for elderly persons’ performance in the WCST was accomplished using the same procedure as Heaton and colleagues,^18,19^ who adopted a method of continuous normalization of results. This procedure was recommended in order to correct any irregularities in score distribution of the variables studied. Heaton and colleagues, by analyzing the distributions of the groups studied, observed that some WCST indicators had sufficiently normal distributions to allow normalizing conversion of the following data: total number of errors, percent errors, perseverative responses, percent perseverative responses, perseverative errors, percent perseverative errors, nonperseverative errors, percent nonperseverative errors and percent conceptual level responses.

For this reason, the results for these technical indicators were selected for continuous normalization using standard-scores (“z”). This presentation model, as proposed by Heaton and colleagues, results in an extensive continuous distribution table of these nine WCST assessment indicators in percentile and other standardized grades. In the present work, in order to summarize this table without compromising its informative value, an eight-interval presentation of the WCST results distribution was elected, corresponding to the test’s diagnosis ranges.

In the process of standardizing the WCST, Heaton and colleagues also identified that another four test indicators were highly asymmetrically distributed, thereby not suggesting its norms in continuous form, from standard-scores. Due to this, the above authors suggested separate handling through categorical normalization for the following technical indicators of the WCST: number of categories completed, trials to complete first category, failure to maintain set and learning to learn.

Thus, elderly AD patient scores for each WCST indicator were distributed into two normative tables, according to their standardized positions. These tables were designed, in the present work, so as to correspond to performance patterns across the specific diagnostic ranges shown in Table 1.

## Results

Table 2 presents descriptive results for the sixteen technical indicators of the WCST, attained by the elderly patients assessed. Both minimal and maximal values are presented, as well as means and standard deviations.

**Table 2:** Descriptive results for assessment indicators of the WCST in elderly persons with AD (n=36).

WCST assessment indicators	Minimum	Maximum	Mean	SD
1. Number of trials administered	128	128	128.00	0.00
2. Total number correct	32	89	56.89	14.32
3. Total number of errors	39	96	71.11	14.32
4. Percent errors	30.47	75.00	55.61	11.25
5. Perseverative responses	12	123	54.25	29.93
6. Percent perseverative responses	9.38	96.09	42.38	23.38
7. Perseverative errors	12	93	44.58	21.00
8. Percent perseverative errors	9.40	72.70	34.84	16.41
9. Nonperseverative errors	0	75	26.53	14.30
10. Percent nonperseverative errors	0.00	58.59	20.84	11.21
11. Conceptual level responses	6	78	35.33	17.36
12. Percent conceptual level responses	5	61	27.61	13.56
13. Number of categories completed	0	3	1.17	0.97
14. Trials to complete first category	10	129	62.31	48.65
15. Failure to maintain set	0	7	1.83	1.87
16. Learning to learn	-36.50	2.80	-19.78	12.33

Table 2 results suggest that these patients were not able to complete all six card classification categories as proposed by the WCST (Number of Categories Completed), having managed no more than three of these. This indicates that, in order to accomplish the test, the patients had to use all 128 available cards (Number of Trials Administered). The mean number of “Total Number Correct” was 56.9, that is, less than half of the cards used. Wrong responses, represented by indicators 3, 4, and 7 to 10, had a mean “Total Number of Errors” of 71.1, chiefly comprising “Perseverative Errors” (44.6), reflecting inflexible and resistant-to-change behavior regarding the task. The other, “Nonpersevarative Errors” (26.5), correspond to exploratory or random response processes during the task.

In summary, Table 2 data shows a low “Total Number Correct” value (56.89), associated to a high proportion of perseverative behaviors (Percent Perseverative Errors, 34.8%) that were inflexible and resistant to change or to mental strategies update, together with a considerable proportion of random or exploratory behaviors (percent nonperseverative errors, 20.8%).

The mean number of “trials to complete first category” (62.3), that is, the number of trials to achieve a “right” in classification of color is high, considering that only 10 trials would be needed to accomplish the whole test. This result shows how difficult the task was, even in its most elementary, first-proposed problem: associate stimuli by their colors. The mean “failure to maintain set” number (1.8) indicates approximately two failures in maintaining attention during the test, thus showing working memory impairment.

The “Learning to learn” indicator (–19.8) assesses the activity learning index during the task. This high, negative value suggests the participants could not effectively learn the task presented by the WCST. This result is coherent with the “percent conceptual level responses” level, which shows only intentional right responses and excludes random “rights”. Only 27.6% of the responses were intentionally correct, which reveals the degree of difficulty in this task for this elderly, clinical group.

A detailed presentation of raw score variability for each WCST assessment indicator is demonstrated in normative Table 3, respecting the classification of eight diagnostic ranges as proposed by the test’s authors.^18,19^

**Table 3:** Preliminary assessment norms for the WCST in elderly persons with initial stage of AD (n=36).

Observed scores									
	Total			Percent		Percent	Non	Percent non	Percent
	number	Percent	Perseverative	perseverative	Perseverative	perseverative	perseverative	perseverative	conceptual level
Diagnostic ranges	of errors	errors	responses	responses	errors	errors	errors	errors	responses
1. Above-average	0-65	0-51	0-39	0-31	0-34	0-27	0-20	0-15	34-100
range									
2. Average range	66-84	52-65	40-81	32-63	35-57	28-44	21-35	16-27	23-33
3. Below-average	85-86	66-69	82-102	64-79	58-77	45-60	36-43	28-33	17-22
range									
4. Mildly impaired	87-94	70-73	103-115	80-90	78-86	61-67	44-74	34-57	6-16
range									
5. Mildly-to-moder-	95-96	74-75	-	-	-	-	-	-	4-5
ately impaired range									
6. Moderately im-	-	-	116-122	91-95	87-92	68-72	-	-	-
paired range									
7. Moderately-to-	-	-	123	96	93	73	-	-	-
severely impaired									
range									
8. Severely impaired	97-128	76-100	124-126	97-100	>93	74-100	75-128	58-100	0-3
range									

The second diagnostic range represents the average performance, on the WCST, of initial-stage AD patients, according to Heaton et al.^19^ The percentiles 29 and 67, that is, the typical response patterns for each assessment indicator in these ranges, suggest that an elderly, initial-stage AD subject is expected to commit 66 to 84 errors during the task, equivalent to more than half the cards being incorrectly classified. Considering perseverative responses, 40 to 81 responses are expected, showing a strong presence of perseverative behavior in these patients’ typical performance pattern. When non-perseverative errors (which are considered exploratory or random) are considered, 35 to 57 responses are expected. Finally, conceptual level responses (intentionally right responses) have an expected 23-33% level for this group.

The first diagnostic range (number 1, above-average range, P≥68) indicates the best performance levels of each WCST indicator. On the other hand, the other ranges (3 to 8) indicate progressive levels of cognitive impairment as evidenced on the task, indicating a below-average performance in the elderly AD group (P≤28).

Considering the differences in age and educational level in the group assessed, we deemed it relevant to analyze the possibility of an interaction of these variables on WCST indicators. To this end, Pearson Correlation calculations (p≤0.05) were performed, and are presented in Table 4.

**Table 4:** Pearson correlation indexes for performance on the WCST in elderly AD patients (n=36), considering age and educational level.

WCST assessment indicators	Age	Educational Level
1. Number of trials administered	-	-
2. Total number correct	-0.11	-0.12
3. Total number of errors	0.11	0.12
4. Percent errors	0.11	0.12
5. Perseverative responses	0.09	0.03
6. Percent perseverative responses	0.09	0.03
7. Perseverative errors	0.10	0.03
8. Percent perseverative errors	0.10	0.03
9. Nonperseverative errors	-0.04	0.07
10. Percent nonperseverative errors	-0.04	0.09
11. Conceptual level responses	-0.13	-0.13
12. Percent conceptual level responses	-0.13	-0.13
13. Number of categories completed	0.15	-0.12
14. Trials to complete first category	-0.13	0.23
15. Failure to maintain set	-0.13	-0.17
16. Learning to learn	-0.03	0.20

The results in Table 4 indicate that age and educational level had near-zero correlation indexes on all 16 assessment indicators of the WCST. None of these reached significant statistical levels (all indexes p=0.05), suggesting the lack of association among these variables and group performance.

## Discussion

Several studies have demonstrated the validity of the WCST in assessing executive functions, as well as its applicability in neuropsychological assessments in general.^20^ At the same time, there are evidences that the task proposed may be very demanding for clinical groups with cognitive impairment signs.^35^ The present study confirmed this tendency, as it evidenced significant impairments in the indicators’ scores of the WCST in elderly, initial-stage AD patients. These performance limitations show the cognitive difficulties of this group in several areas: concept formation, working memory, planning, cognitive flexibility, self-monitoring, perseverative behavior inhibiting and learning, as expected, according to the WCST’s original purpose.^19^

As AD is associated to degenerative processes of both brain structures and functionality, we expected, in the present work, poorer performance on the WCST, reflecting real cognitive difficulties experienced by patients. Therefore, these results constitute empirical evidences in support of the test’s clinical validity.

Also regarding the general pattern of low results on the WCST - showing functional impairment of the group’s executive processing - it was possible to discriminate among differential performance patterns, allowing identification of higher or lower cognitive loss. Moreover, the variability of the results made enabled different executive impairment levels to be identified, thus allowing WCST performance categorization into eight diagnostic ranges, as predicted. These represent average performance and its possible deviations, suggesting progressive impairment levels on the task.

Although age and educational level could be considered protective factors for cognitive decline in dementia,^36^ the present work did not observe an association of these variables and WCST performance in AD patients. Even when considering age and educational level variations (62 to 86 years’ old, M=75.8, and 1 to 12 years of schooling, M=4.9, respectively), there were no discriminative advantages or disadvantages for the participants. Despite the preliminary nature of the present study coupled with the small sample - which certainly did not correspond to the full cognitive variability of AD - the data in the present study suggests a direct relationship between WCST performance and this clinical condition. Such data can offer relevant information for the diagnosis of this kind of executive impairment in the elderly.

The present study also allowed preliminary normative standards to be devised for elderly AD patients on the WCST, which offer updated resources for both analyzing the characteristics of other same-condition individuals’ performance or even detecting AD before it appears. Thus, it was possible to devise a relevant technical reference for neuropsychological assessment in the Brazilian context. Future interpretation of individual results based on these preliminary norms allows patient performance categorization - for each assessment indicator of the test - into diagnostic ranges which can identify higher or lower executive function impairment, as well as any problematic areas, compared to this reference group.

A limitation of this study was the small sample assessed, which does not cover the very elderly and AD’s clinical condition. Ideally, the actual results of the WCST in the AD group should be compared with healthy elderly subjects. A comparative investigation of this nature could allow the test’s discriminative validity for AD to be verified, through technical appraisal of the best indicators for this, as well as permitting adequate cut-off points for each to be identified. Similar lines of investigation have been developed by several Brazilian research centers, producing interesting results.^25^ However, the non-existence of assessment parameters for elderly AD patients’ executive functions prior to the present study, lends weight for the presentation of these current, promising results.

However, it is important to stress that no psychological assessment instrument can, alone, adequately represent reality and human complexity. Parsimonious interpretations of results are essential for any psychological technique, including the WCST, where such instruments must be considered complementary elements for each clinical case assessment, requiring other technical and investigative resources in order to understand both AD’s and individuals’ complexity.
